# Pharmacological and genetic manipulations at the µ-opioid receptor reveal arrestin-3 engagement limits analgesic tolerance and does not exacerbate respiratory depression in mice

**DOI:** 10.1038/s41386-021-01054-x

**Published:** 2021-07-13

**Authors:** Li He, Sarah W. Gooding, Elinor Lewis, Lindsey C. Felth, Anirudh Gaur, Jennifer L. Whistler

**Affiliations:** 1 Center for Neuroscience, University of California–Davis, Davis, CA USA; 2 Department of Physiology and Membrane Biology, UC Davis School of Medicine, Davis, CA USA

**Keywords:** Neuroscience, Cellular neuroscience

## Abstract

Opioid drugs are widely used analgesics that activate the G protein-coupled µ-opioid receptor, whose endogenous neuropeptide agonists, endorphins and enkephalins, are potent pain relievers. The therapeutic utility of opioid drugs is hindered by development of tolerance to the analgesic effects, requiring dose escalation for persistent pain control and leading to overdose and fatal respiratory distress. The prevailing hypothesis is that the intended analgesic effects of opioid drugs are mediated by µ-opioid receptor signaling to G protein, while the side-effects of respiratory depression and analgesic tolerance are caused by engagement of the receptor with the arrestin-3 protein. Consequently, opioid drug development has focused exclusively on identifying agonists devoid of arrestin-3 engagement. Here, we challenge the prevailing hypothesis with a panel of six clinically relevant opioid drugs and mice of three distinct genotypes with varying abilities to promote morphine-mediated arrestin-3 engagement. With this genetic and pharmacological approach, we demonstrate that arrestin-3 recruitment does not impact respiratory depression, and effective arrestin-3 engagement reduces, rather than exacerbates, the development of analgesic tolerance. These studies suggest that future development of safer opioids should focus on identifying opioid ligands that recruit both G protein and arrestin-3, thereby mimicking the signaling profile of most endogenous µ-opioid receptor agonists.

## Introduction

Opioids are highly effective analgesics and are essential for the treatment of severe pain [1]. However, prolonged use produces analgesic tolerance, necessitating dose escalation and increasing the risk of respiratory arrest [2]. Incidence of opioid overdose has climbed for decades and accounts for thousands of deaths by respiratory distress annually [3]. Recently, the charge to develop safer analgesic therapies has prioritized ameliorating the respiratory side-effects of opioid drugs.

Both the analgesic and respiratory effects of opioids are primarily mediated through the µ-opioid receptor (MOR) [4, 5], a G protein-coupled receptor (GPCR). GPCRs, when bound by agonist, activate heterotrimeric G proteins. Arrestin-3 (β-arrestin-2) titrates G protein signaling from GPCRs by promoting desensitization and endocytosis of receptors and scaffolds other signal effectors [6]. GPCR agonists can vary in their signaling bias: the relative engagement of G protein versus arrestin-3 recruitment [7].

Opioid drugs vary in both their degree of signaling bias and effect/side-effect profile [8]. This has inspired hypotheses as to the role of signaling bias in their therapeutic utility. The predominant hypothesis that G protein signaling promotes the analgesic effects of opioids, while arrestin-3 engagement is detrimental and causes the respiratory depressive side-effects, arose from the finding that mice lacking arrestin-3 (Arr-3 KO) displayed reduced respiratory depression in response to morphine (a MOR agonist) [9]. This finding led to a decade-long drive toward the development of MOR agonists devoid of arrestin-3 engagement with a goal to abolish respiratory depression while maintaining analgesia. However, most endogenous ligands efficiently engage arrestin-3 [10], and the small molecule opioid drug methadone, which also strongly engages arrestin-3, produces less tolerance and dependence than morphine [11], suggesting an alternative strategy for the development of safer opioids [12]. To clarify the role of arrestin-3 in the opioid side-effects of respiratory depression and tolerance, we examined six clinically relevant opioids with varying G protein biases at equi-analgesic doses. We also examined the respiratory side-effects of morphine in mice with varying abilities to recruit arrestin-3 to morphine-bound MOR: wild type (WT) mice, Arr-3 KO mice and a mouse expressing a mutant MOR (RMOR, for recycling MOR) that engages both G protein and arrestin-3 in response to morphine [13]. RMOR’s arrestin-3 engagement reduces the side-effects of opioid use that drive dose escalation, including tolerance [14] and abuse liability [15]. Here, we examine respiratory side-effects in these mice and show that arrestin-3 engagement at MOR does not exacerbate respiratory depression and protects against the development of analgesic tolerance. This suggests the search for safer opioids should focus on identifying drugs that engage arrestin-3 to avoid the development of tolerance and lower abuse potential.

## Methods

### Drugs

[D-Ala^2^, N-Me-Phe^4^, Gly^5^-ol]-Enkephalin acetate salt (DAMGO), (±)-methadone hydrochloride, fentanyl citrate, buprenorphine hydrochloride, oxycodone hydrochloride and naloxone hydrochloride dihydrate were purchased from Sigma (St. Louis, MO), morphine sulfate was purchased from Mallinckrodt (St. Louis, MO) and TRV130 hydrochloride was purchased from Adooq Biosciences LLC (Irvine, CA). The drugs were dissolved in physiological saline for in vivo studies except TRV130, which was dissolved in physiological saline with a final concentration of 1.5% DMSO for in vivo studies. All drugs were administered s.c. to mice in a volume of 10 ml/kg. For in vitro pGlo studies, drugs were dissolved in deionized water or 1.5% DMSO to a concentration of 10 mM and further diluted to obtain concentrations between 0.5 nm and 50 μM. No difference was found between 1.5% DMSO and deionized water. For in vitro arrestin-3 recruitment studies, all drugs were diluted in 1% DMSO with deionized water.

### Tail-flick assay

Analgesia was measured using a cumulative dose–response in the radiant heat tail-flick assay (Tail-flick Analgesia Meter, Columbus Instrument. Columbus, OH). The intensity of the light was adjusted so that baseline tail-flick latencies ranged from 1.4 to 2.0 s, and a cutoff time of three times the mean baseline latency was set to minimize damage to the tail. Each group consisted of at least 8 mice. Analgesia was tested 20 min after each s.c. administered dose, except with TRV130, which was tested after 10 min. Three to four doses per mouse were used to establish the dose–response curve for each drug. Data are displayed as the “maximum possible effect” (%MPE): 100*[(drug response time − baseline latency)/(cutoff time − baseline latency)]. Cumulative dose–response curves were fit using GraphPad Prism, and the AD_50_ values of the opioid drugs and their 95% confidence intervals were calculated. The AD_80_ values for each drug were calculated from the same curves. To measure tolerance, mice were administered the drugs at the AD_80_ for 6 days following a cumulative dose–response test on day 1. On Day 8, a second cumulative dose–response and AD_50_ was determined and a fold shift in AD_50_ from Day 1 to Day 8 was calculated.

### Withdrawal assessment

WT C57BL/6 and RMOR mice were treated with morphine (8 mg/kg and 1.5 mg/kg, respectively) once daily for 6 days. Thirty minutes after the final drug injection, mice were injected with naloxone (5 mg/kg, s.c.) and observed for 20 min by an observer who was blind to experimental conditions. Standard withdrawal behaviors were used to score each mouse and included wet-dog shakes, jumps and paw tremors.

### Mouse plethysmography

Respiration data was collected using a whole-body plethysmography system (Data Sciences International (DSI), St. Paul, MN). Mice were placed in the chambers after DSI airflow transducers were attached to each plethysmography chamber to maintain a constant flow rate. Each chamber was calibrated to its attached transducer before recording. Mice were habituated to the clear plexiglass chambers for 20 min per day for two days. On Day 3, mice were first habituated to the chambers for 10 min. Respiratory parameters were then recorded for 10 min to establish a baseline before injection of vehicle or drugs and were collected for 80 min post drug injection at 10 min intervals. Opioid drugs were given to mice at their AD_80_ analgesic dose to measure their respiratory depressive effects. To measure respiratory tolerance, mice were administered the AD_80_ dose for 6 days following the first testing day. On Day 8, mice were given the AD_80,_ and their respiratory rates were measured as on Day 1. Both area under the curve (AUC) and maximum respiratory depression were  calculated (minimum respiratory rate/baseline rate x 100 for each mouse.)

### Arrestin-3 recruitment assay

CHO-K1 OPRM1 PathHunter® β-arrestin-2 cells (DiscoverX, Fremont, CA, USA) were seeded (2500 cells per well) in a low-volume, round-bottom, opaque 384-well plate. The next day, the cells were stimulated for 90 min with a series of MOR agonists (3 nM–30 μM) in 1% DMSO (at 37 °C/5% CO_2_.) Arrestin-3 recruitment was detected following a 60 min incubation period, according to the manufacturer’s guidelines, with a PathHunter Detection reagent. Luminescence was measured using a Flexstation3 (Molecular Devices, San Jose, CA, USA). EC_50_ and *E*_max_ values were determined with a nonlinear fit using GraphPad Prism 8 software (GraphPad Software, La Jolla, CA), and data were normalized and plotted as % *E*_max_ produced by DAMGO. Each drug was tested in a minimum of three independent experiments with all doses in quadruplicate. Cells are tested monthly for mycoplasma.

### Statistics

Respiratory effects (maximum respiratory depression and AUC) for each genotype at equi-analgesic doses were compared in both saline and morphine conditions using a two-way ANOVA with Tukey’s multiple comparisons test. The effects of different morphine doses in 129SvJ mice were also compared to saline using a one-way ANOVA with Dunnett’s multiple comparisons test. The respiratory effects of the opioid drug panel were assessed by comparing all drug conditions using a one-way ANOVA with Dunnett’s multiple comparisons test. To assess respiratory tolerance, respiratory rates were normalized as % baseline to account for baseline drift over time, and within-subjects comparisons for each drug on Day 1 and Day 8 were made using a two-way ANOVA with repeated measures and Sidak’s multiple comparisons test. For in vitro *assays*, IC_50_, EC_50_ and *E*_max_ values for each drug were compared to the effect of DAMGO using a one-way ANOVA with Dunnett’s multiple comparisons test. Arrestin-3 recruitment or cAMP inhibition were correlated with respiratory depression, analgesia or analgesic tolerance (calculated as fold shift in analgesic AD_50_ from day 1 to day 8) using a simple linear regression.

### Mice

Male C57BL/6 mice were purchased from Charles River Laboratories (Wilmington, MA), and male 129SvJ mice were purchased from The Jackson Laboratory (Sacramento, CA). Mice acclimated to the housing conditions for at least 5 days after arriving and were maintained under a standard 12/12-h light/dark cycle, with ambient temperature set at 20 ^o^C to 22 ^o^C and food and water ad libitum. Recycling MOR (RMOR) [11] mice have been bred congenic to C57BL/6 for >30 generations. Arrestin-3 (Beta arrestin-2) KO mice were generously provided by Dr. R. Lefkowitz (Duke University) and have been bred congenic to C57BL/6 for >30 generations. Mice aged 12–18 weeks were used and housed in groups of 3–4.

### Study approval

All protocols were approved by the Institutional Animal Care and Use Committee at University of California Davis and are in accordance with the National Institutes of Health guidelines for care and use of laboratory animals

## Results

### Arrestin-3 engagement improves morphine-induced analgesia and reduces morphine tolerance

**Fig. 1:**
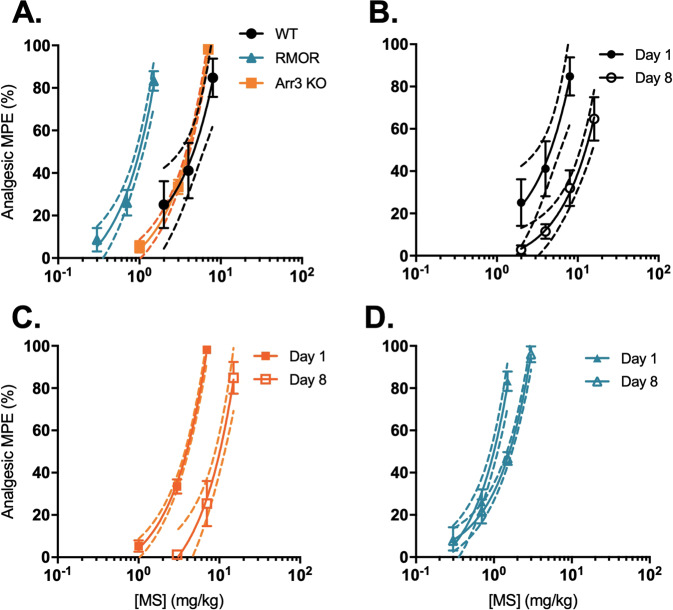
Morphine analgesia and tolerance varies with arrestin-3 recruitment. **A** Acute morphine (MS) analgesia in WT mice (*N* = 8, black), Arr-3 KO mice (*N* = 7, orange) and RMOR mice (*N* = 8, blue). **B**–**D** Tolerance: analgesic effects of MS on Day 1 (closed symbols) and Day 8 (open symbols) in (**B**) WT mice, (**C**) Arr-3 KO mice and (**D**) RMOR mice. Mice received an AD_80_ dose of MS on days 2–7. RMORs showed reduced tolerance compared to WT and Arr-3 KO mice. Error bars represent SEM. Dotted lines represent 95% confidence intervals of the linear regression. Shift in EC_50_ from Day 1 to Day 8 shown in Table S1.

We measured morphine-induced analgesia with a radiant heat tail-flick assay in three different mouse genotypes: WT, in which MOR poorly engages arrestin-3 in response to morphine [14], Arr-3 KO [16] and knock-in mice with a mutant MOR that robustly recruits arrestin-3 (RMOR) [13]. Using a cumulative dosing regimen, we found that WT and Arr-3 KO mice exhibit a similar analgesic dose–response to morphine (AD_50_ of 4.6 and 3.9 mg/kg, respectively) (Fig. 1A and Table S1). However, as previously reported [14], RMOR mice show enhanced morphine analgesia compared to WT (AD_50_: 1.0 mg/kg) (Fig. 1A and Table S1). This suggests morphine analgesia is improved rather than impaired by engagement of arrestin-3 at MOR.

We next examined how arrestin-3 recruitment alters analgesic tolerance to morphine. The AD_80_ dose of morphine for each genotype was calculated using the Day 1 dose–response (Fig. 1A) and was administered to the mice daily for 6 days (8, 6, and 1.5 mg/kg in WT, Arr-3 KO and RMORs, respectively, s.c.). On Day 8, we performed a second dose–response and calculated the shift in morphine potency for each genotype (Fig. 1B, C, D and Table S1). Both WT and Arr-3 KO mice showed a rightward shift in their dose–response curves (AD_80_ fold shift 2.7 and 2.6, respectively), indicating development of analgesic tolerance as higher doses of morphine were required on Day 8 to achieve the same %MPE (Fig. 1B,  C and Table S1). RMOR mice exhibited reduced analgesic tolerance compared to WT (Fig. 1D and Table S1), expanding on previous reports [14] by showing no shift in the cumulative dose–response curve to morphine in RMOR mice.

### Arrestin-3 engagement does not exacerbate respiratory depression

**Fig. 2:**
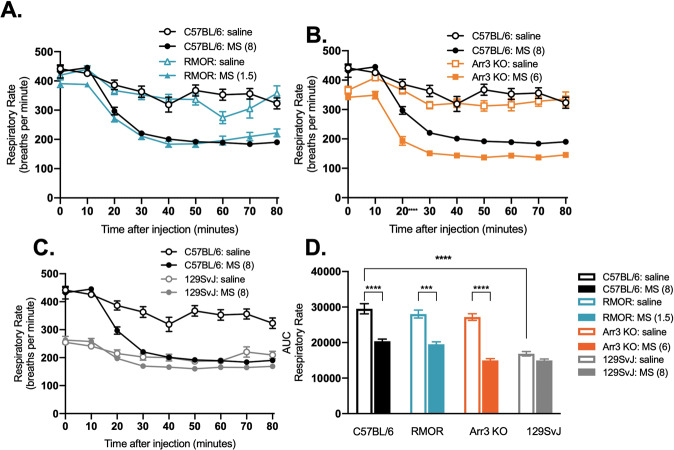
Morphine-induced respiratory depression is independent of arrestin-3 recruitment and is dependent on genetic background. **A** Respiratory depression in C57BL/6 WT mice (black, *N* = 8) and RMOR mice (*N* = 8, blue) at the AD_80_ equi-analgesic dose determined in Fig. 1. **B** Respiratory depression in C57BL/6 WT mice (black, *N* = 8) and Arr-3 KO mice (*N* = 8, orange) at the AD_80_ equi-analgesic dose determined in Fig. 1. **C** Respiratory depression in C57BL/6 WT mice (*N* = 8, black) and 129SvJ WT mice (*N* = 8, gray) given equivalent morphine. **D** Area under the curve for **A**–**C** (see Table S1). Error bars represent SEM.

Previous studies report that loss of arrestin-3 engagement, either using Arr-3 KO mice [9] or novel, putative G-biased MOR agonists, reduces opioid-induced respiratory depression [17–19] although that has been recently challenged [20, 21]. This implied that enhanced arrestin-3 recruitment, as occurs in RMOR mice, should increase respiratory depression. We assessed respiratory depression with whole-body plethysmography in WT, Arr-3 KO and RMOR mice in response to the AD_80_ dose of morphine for each genotype. RMOR and WT mice showed comparable respiratory depression in response to equi-analgesic morphine (Fig. 2A, D and Table S1), indicating arrestin-3 engagement does not exacerbate respiratory depression. In contrast to the original report [9], Arr-3 KO also showed equivalent respiratory depression compared to WTs (Fig. 2B, D and Table S1), supporting recent reports from three independent laboratories [22].

This discrepancy in phenotype may be due to the mixed C57BL6/129SvJ background of the mice used in the original study [9, 16]. The Arr-3 KO mice used in this study, although originally obtained from the same source, have been bred congenic to C57BL/6 for >30 generations. 129SvJ mice are resistant to analgesic morphine tolerance [23, 24] and differ in several other physiological responses to morphine and other drugs of abuse [25–29]. We found that 129SvJ mice have a significantly lower baseline respiratory rate (254.7 ± 9.4 breaths/min) than C57BL/6 (441.5 ± 10.2 breaths/min) (*F*_(3,66)_ = 25.41, *P* < 0.0001 in a two-way ANOVA with Tukey’s multiple comparisons test) and do not show morphine-induced respiratory depression at the equi-analgesic dose (8 mg/kg) of C57BL/6 (Fig. 2C and Table S2). We also measured respiratory depression in 129SvJ mice with 50 mg/kg morphine and found that it did significantly reduce respiratory rate (Ordinary one-way ANOVA, *F* = 20.79, *P* < 0.0001) (Fig. S1), suggesting that 129SvJ resistance to morphine’s respiratory effects results from lower sensitivity than C57BL/6, rather than a prohibitive effect from the lower baseline respiratory rate (Fig. S1). These results support the possibility that differences between historical and contemporary studies of Arr-3 KO mice could be explained by genetic background.

**Fig. 3:**
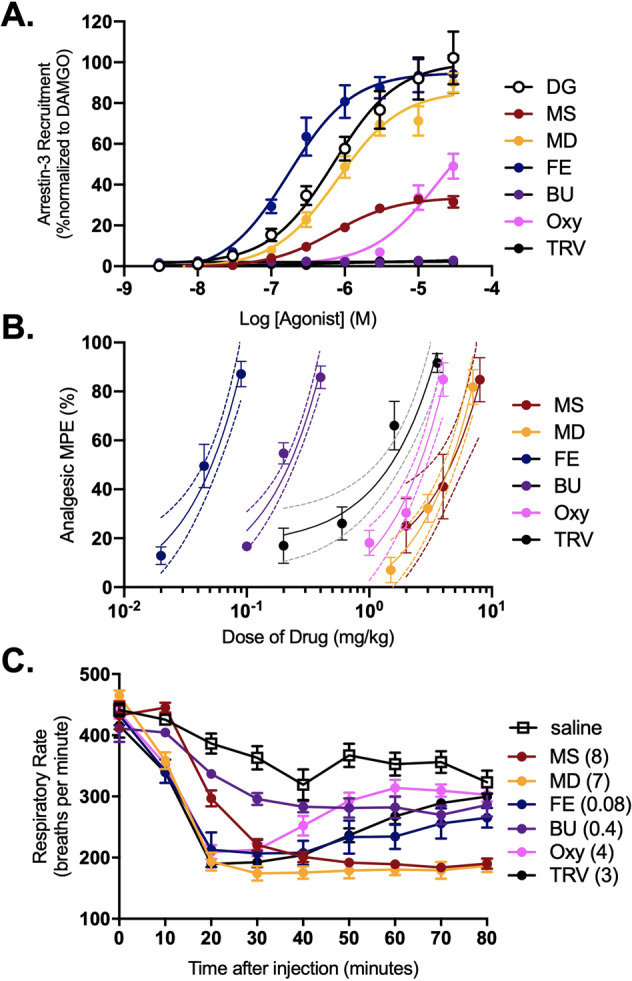
Clinically important opioid drugs with variable arrestin-3 engagement produce respiratory depression at equi-analgesic doses. **A** Arrestin-3 recruitment in MOR PathHunter arrestin-3 cells in response to DAMGO (DG), morphine (MS), methadone (MD), buprenorphine (BU), fentanyl (FE), oxycodone (OXY) and TRV130 (TRV). Data points for TRV and BU overlap at several points. EC_50_ and *E*_max_ values are shown in Table S3. Data are mean +/− SEM from 3 to 7 experiments, each performed in quadruplicate. **B** Analgesic dose–response to each drug in C57BL/6 WT mice (*N* = 8–10). **C** Respiratory depression to the AD_80_ analgesic dose of each agonist (*N* = 8–10). AD_50_ and Area Under the Curve (AUC) for **B** and **C**, respectively, are shown in Table S4. Peak (maximum respiratory depression) data for **B** is shown in Table S5. Maximums were compared to all other drugs with an ordinary one-way ANOVA and Tukey’s multiple comparisons test (ns). Error bars represent SEM. Dotted lines represent confidence intervals calculated from the curve fit.

We then compared the abilities of five MOR agonist drugs (morphine, methadone, fentanyl, buprenorphine, and oxycodone), as well as the recently FDA-approved TRV130 (oliceridine) [19], to engage arrestin-3 using the DiscoverX Pathhunter assay. We found that all drugs tested exhibited lower *E*_max_ arrestin-3 recruitment than [D-Ala^2^, N-Me-Phe^4^, Gly^5^-ol]-Enkephalin (DAMGO, a hydrolysis-resistant form of enkephalin), with methadone (86.0%) and fentanyl (94.9%) the most similar, followed by oxycodone and morphine (78.9% and 33.8%, respectively), while TRV130 and buprenorphine showed negligible arrestin-3 engagement (Fig. 3A and Table S3).

The analgesic potency of each drug was determined in WT C57BL/6 mice using a cumulative dose–response protocol (Fig. 3B and Table S4). DAMGO was excluded because it cannot be administered systemically; it does not, however, produce analgesic tolerance when administered intrathecally [30]. We then administered the AD_80_ dose of each drug and assessed degree of respiratory depression (Fig. 3D). We found that morphine, methadone, fentanyl, oxycodone and TRV130 did not significantly differ in their maximum respiratory depression in an ordinary one-way ANOVA with Tukey’s multiple comparisons test (Figs. 3C and Table S5), but these drugs showed variable kinetic effects with regards to onset and duration of respiratory depression. The acute respiratory effect of buprenorphine administered at the AD_80_ dose was not significantly different from saline (Fig. 3C). These results, together with those from Fig. 2, indicate no correlation (Figs. 5A and S2A) between magnitude of arrestin-3 recruitment and degree of respiratory depression.

### Correlation analysis of in vitro signaling properties and in vivo side-effects reveals an inverse correlation between arrestin-3 engagement and analgesic, but not respiratory tolerance

**Fig. 4:**
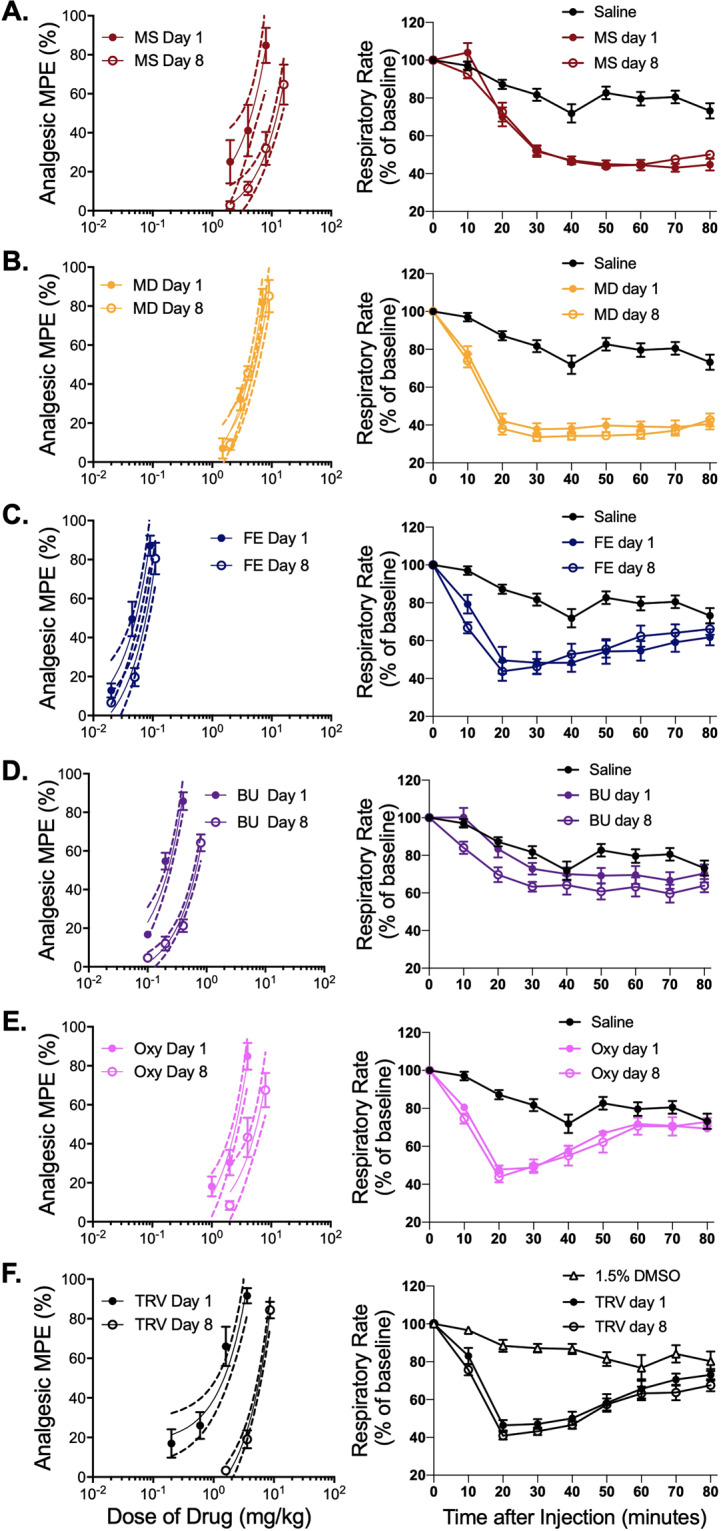
Analgesic tolerance is dependent on degree of arrestin-3 recruitment. No respiratory tolerance develops to these drugs. (**A**–**F**, left panels). Analgesia to each drug (**A**–**F** left panels, *N* = 8 per drug) was measured on day 1 (closed symbols) and day 8 (open symbols). Mice were given the analgesic AD_80_ dose of drug on days 2–7. (**A**–**F**, right panels) The respiratory depressive effects of each drug (*N* = 8 per drug) were measured on day 1 (closed symbols) and day 8 (open symbols). Mice were given the analgesic AD_80_ dose of drug on days 2–7. Day 1 and 8 are shown as percent of baseline for each drug. AD_50_ values for analgesia and area under the curve (AUC) for respiration on Day 1 and Day 8 are shown in Table S4. **A** morphine, **B** methadone, **C** fentanyl, **D** buprenorphine, **E** oxycodone, and **F** TRV130. All drugs, except methadone, produced significant analgesic tolerance. No drug produced respiratory tolerance. Error bars represent SEM. Dotted lines represent confidence intervals calculated from the curve fit.

Analgesic tolerance following repeated use of opioids necessitates dose escalation for persistent pain control [31]. We assessed analgesic tolerance to the AD_80_ dose of each drug in our panel in WT mice with the protocol used for morphine in Fig. 1. Mice showed analgesic tolerance to all drugs except methadone (Fig. 4 left panels, Table S4). In a separate cohort, we assessed respiratory tolerance by measuring respiration in response to the AD_80_ dose of each drug on Day 1 and Day 8 after daily treatment with the AD_80_ dose (Fig. 4 right panels, Table S4). Each drug’s respiratory effect on Day 1 and Day 8 was compared, and the same dosing regimen that produced analgesic tolerance did not cause tolerance to the respiratory depressive effects of any of these drugs (no significance confirmed with two-way RM ANOVA with Sidak’s multiple comparisons test).

**Fig. 5:**
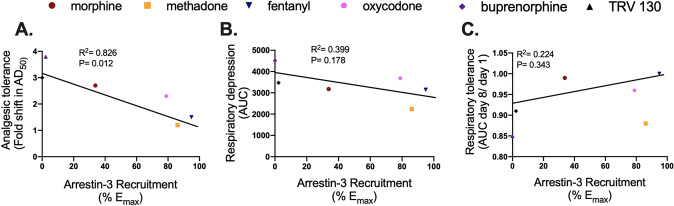
Analgesic tolerance but not respiratory depression correlates with arrestin-3 recruitment. **A** Correlation analysis between the *E*_max_ of arrestin-3 recruitment (Fig. 3A) and analgesic tolerance to each drug (Fig. 4A–F left panels, Table S4). There is a significant inverse correlation where increased arrestin-3 engagement correlates with reduced analgesic tolerance. **B**, **C** Correlation analysis between the *E*_max_ of arrestin-3 recruitment (Fig. 3A) and tolerance to the respiratory effects of each drug (Fig. 4A–F right panels, Table S4).

We next correlated the in vitro arrestin-3 recruitment with the degree of analgesic tolerance to each drug (Figs. 5C and S2E). This analysis revealed a strong negative correlation between arrestin-3 recruitment and the development of analgesic tolerance (*R*^2^ = 0.829, *P* = 0.012). Drugs that efficiently recruit arrestin-3 at MOR promote low analgesic tolerance, and poor arrestin-3 recruiters cause the most analgesic tolerance. These results complement those in Fig. 1 showing that RMOR mice, in which arrestin-3 is engaged by morphine, are resistant to analgesic tolerance. These data suggest that arrestin-3 recruitment improves the therapeutic utility for opioids by reducing analgesic tolerance so that dosage may remain low enough to mitigate respiratory risks.

## Discussion

The experiments herein show that WT, Arr-3 KO and RMOR mice have similar respiratory responses to morphine (Fig. 2), and the promotion of arrestin-3 recruitment by six opioids at equi-analgesic doses is unrelated to their respiratory effects (Figs. 3, 5A and S2A). These results suggest that arrestin-3 engagement is not a predictor of respiratory risk for opioids. We further demonstrate that arrestin-3 engagement in RMOR mice treated with morphine and WT mice treated with methadone reduces the development of analgesic tolerance, indicating a novel therapeutic strategy for opioid analgesic drug development that prioritizes engagement of arrestin-3. Our results complement recent studies from several groups reporting no difference in respiratory depression between WT and Arr-3 KO mice in response to morphine or fentanyl [22]. Another genetically modified mouse, which lacks phosphorylation of MOR (a phospho-null mouse) and consequently, arrestin-3 recruitment, also does not show improved respiratory depression [32]. Our finding that 129SvJ mice are less sensitive to morphine-induced respiratory depression suggests the likelihood that the early Arr-3 KO respiratory phenotype was affected by genetic background. Furthermore, a growing body of evidence suggests that G protein mechanisms, such as GIRK channels [33–35] and Gɑ_i_ signaling [36], are responsible for opioid-induced respiratory depression, and low G protein efficacy explains the reduced respiratory effect observed with the newer ultra-biased ligands [20]. All of this implicates G protein activity over arrestin-3 as the key player in opioid-induced respiratory depression, leaving no clear way to separate the analgesic and respiratory mechanisms of these drugs. Therefore, it is critical to investigate mitigating strategies that consider factors beyond respiratory pathways, such as tolerance or abuse liability. It is also important to keep in mind that each of these drugs has its own unique profile with significant variability in half-life, intrinsic efficacy, affinity and off-target effects, all of which can complicate interpretation of in vivo data.

### Tolerance changes the therapeutic utility of morphine

Analgesic tolerance to opioids drives dose escalation, which increases respiratory risk. Tolerance to the respiratory effects of opioids develops at a slower rate than analgesic tolerance, shrinking therapeutic utility, as the higher doses needed to maintain analgesia approach levels that risk respiratory distress [37–41]. Our results are consistent with these observations.

While arrestin-3 recruitment at MOR appears unrelated to respiratory depression (Figs. 5A, B and S2A), we found a strong negative correlation between the development of analgesic tolerance in vivo and arrestin-3 recruitment in vitro using a diverse panel of opioids. Of course, as mentioned above, these drugs vary in several properties beyond arrestin-3 recruitment, including affinity, intrinsic efficacy, bioavailability, half-life and off-target effects. TRV130, for example, has a shorter lasting analgesic effect [19] and a more rapid return to normal breath rate than the longer acting drugs (so a smaller area under the curve), although acute respiratory depression in our hands is equivalent to that produced by morphine where the ED_80_ dose of each drug was chosen based on a cumulative dose–response for analgesia in the tail-flick assay. In this paradigm, 3 mg/kg of TRV130 was equivalent to 8 mg/kg of morphine for analgesia. This 3 mg/kg dose of TRV130 showed comparable respiratory depression to 8 mg/kg of morphine, which is similar to what was initially reported [19] but is higher than that shown in another recent report, which that measured analgesia with the hot plate assay [20]. More importantly, despite its short duration of action, TRV130 produced the greatest analgesic tolerance among the drugs we tested. For example, buprenorphine showed the lowest degree of respiratory depression, but its effect was more prolonged than that of TRV130, perhaps reflecting their differences in binding kinetics [42]. Note that, drugs with both long (morphine) and short (oxycodone) duration of action produced tolerance when given in doses equi-analgesic for their acute effect.

Differences in ligand efficacy for G protein in particular, are likely critical to the interpretation of data comparing different drugs. Recently, it was shown that several putative G protein-biased ligands are partial agonists for G protein signaling in non-amplified systems and/or where receptor number is limited, and therefore are not, in fact, G biased [20]. This includes TRV130, PZM-21 and SR17018. The authors demonstrate that the low intrinsic efficacy of these more recent agonists is causal to their reduced respiratory depression (see refs. [21, 43] and references therein). For analgesic tolerance liability, ligand efficacy could likewise be an important property. However, even with the more sensitive efficacy assays employed in the above studies, methadone showed intrinsic efficacy comparable to DAMGO and higher than morphine and oxycodone. Nevertheless, methadone did not produce tolerance at equi-analgesic doses to morphine (Fig. 4B), suggesting that mechanisms other than intrinsic efficacy are at play.

To circumvent the variable of intrinsic efficacy (as well as different pharmacokinetics or other ligand properties), we used RMOR mice to show that tolerance to morphine is reduced by enhancing arrestin-3 engagement by the receptor without any increase in the respiratory depression (Fig. 2A). The RMOR receptor was created by substituting a portion of the cytoplasmic tail of MOR with that of DOR (δ-opioid receptor), creating a receptor that is a better substrate for G protein coupled receptor kinases [44] without altering ligand affinity [14]. We cannot rule out that this substitution alters signaling in some way other than by increasing engagement of arrestin-3. However, the efficacies of morphine and DAMGO for G protein signaling ex vivo are indistinguishable from the WT MOR in the VTA for both GIRK signaling and effects on GABA release [45]. One additional way to selectively examine the effects of arrestin-3 engagement versus efficacy on tolerance would be to examine the effects of methadone (which promotes arrestin-3 engagement and has G protein efficacy comparable to DAMGO [20]) in the phospho-null mice [32]. Furthermore, it would be interesting to examine the effects of SR17018 in the phospho-null mice. Grim et al. posit that the reduced tolerance to SR17018 is a function of poor arrestin-3 recruitment [46]. However, Gillis et al. [20] show that SR17018 is not G protein-biased but rather shows low efficacy for both G and arrestin-3 (therefore “balanced”). If arrestin-3 recruitment reduces tolerance, we would expect methadone and SR17018 to produce more tolerance in the phospho-null mice compared to WT mice. If arrestin-3 engagement produces tolerance, the phospho-null mice should show reduced tolerance. In our hands, MOR engagement of arrestin-3 in RMOR mice did not exacerbate respiratory side-effects and improved analgesic efficacy during prolonged treatment by preventing the development of analgesic tolerance. Our results suggest that agonists that engage both G protein and arrestin-3 have the potential to improve the therapeutic utility of opioids, maintaining analgesic potency through prolonged administration without increasing respiratory risk. It may be that optimizing both for “balanced” signaling and lower efficacy could greatly improve opioid safety.

### Arrestin-3 can influence tolerance through multiple mechanisms

Enhanced arrestin-3 binding (RMOR mice) [14] and arrestin-3 recruitment by methadone [11] protect against analgesic tolerance, and endocytosis of MOR is associated with a reduction in analgesic tolerance [47, 48]. Previous studies have also reported reduced morphine tolerance in Arr-3 KO mice [49] and phospho-null mice that lack arrestin-3 binding [32]. We found that Arr-3 KO mice had a small but not significant decrease in analgesic tolerance compared to WT, which could reflect a difference in analgesic assays between studies or the aforementioned differences in genetic background. We propose a model to reconcile reduced tolerance in these three different mouse models by explaining how either elimination or enhancement of arrestin-3 activity could decrease tolerance.

Both desensitization of the receptor on the cell membrane and homeostatic adaptations that oppose the MOR signaling cascade produce analgesic tolerance to opioids. Compared to endogenous ligands, morphine produces incomplete phosphorylation of receptor [50, 51], poor arrestin-3 recruitment [52, 53], partial desensitization [54] and poor resensitization [55], especially following multiple drug exposures [55, 56]. This rapid, partial and largely irreversible desensitization of MOR in response to morphine is likely reduced in mice lacking all MOR phosphorylation sites [32] or lacking arrestin-3 [16]. By extension, we would expect such mice to show enhanced analgesia to acute morphine. Rapid resensitization, as occurs with endorphins in WT mice or morphine in RMOR mice, would also be expected to enhance analgesia. In fact, Arr-3 KO, phospho-null and RMOR mice all exhibit enhanced acute morphine analgesia [14, 16, 32]. We hypothesize that partial, irreversible receptor desensitization plays a minor role in the development of analgesic tolerance, which is primarily driven by homeostatic adaptations downstream of the receptor that occur when G protein signaling from MOR is not properly titrated by arrestin-3 engagement and the cycle of desensitization and resensitization. This model predicts that both Arr-3 KO and phospho-null mice would show reduced morphine tolerance compared to WT (or to any drug that produced partial irreversible phosphorylation/desensitization). Also, more balanced agonists may cause increased tolerance in these strains as the absence of arrestin-3 activity would promote the homeostatic adaptations that morphine produces in WT mice, a hypothesis that has not yet been tested. This model also predicts that RMOR mice would exhibit even less tolerance by preventing both partial desensitization and mitigating the homeostatic adaptations occurring downstream of the receptor [45, 57]. This is completely consistent with our data in Fig. 1. By extension, agonists that properly titrate G protein signal by promoting full phosphorylation of receptors, arrestin-3 engagement, receptor endocytosis and recycling would show excellent analgesia and reduced tolerance.

### Arrestin-3 engagement may reduce abuse potential of opioids

Few studies have addressed the role of arrestin-3 recruitment in abuse potential. Opioid abuse is frequently a consequence of dose escalation due to analgesic tolerance, but the abuse liability of opioids is exacerbated by physical dependence and withdrawal symptoms when the drug is removed. Previous reports show that disruption of arrestin-3, either genetic or pharmaceutical, has no effect on the development of morphine dependence [32, 49, 58]. In contrast, compared to WTs, RMOR mice show attenuated morphine dependence [14], indicated by reduced withdrawal behaviors after 8 days of morphine treatment (Fig. S3). RMOR mice also show reduced affective dependence, assessed by conditioned place aversion to naloxone after repeated morphine treatment [15].

While reward is a popular proxy for abuse liability, more study on the relationship between arrestin-3 engagement and opioid reward is necessary. While both RMORs [15] and Arr-3 KOs [59] show enhanced morphine conditioned place preference (CPP), RMOR mice, unlike WT, do not transition to compulsive drug seeking in self-administration paradigms or show reinstatement to drug seeking after extinction [15], demonstrating that reward is a flawed indicator of abuse potential. Furthermore, while both PZM-21 and TRV130, which promote negligible arrestin-3 engagement, show reduced CPP compared to morphine [18, 58], TRV130 is comparable to other opioids in its promotion of self-administration, facilitation of intracranial self-stimulation and physical dependence [58, 60, 61]. The current evidence, therefore, favors a high abuse liability for agonists that do not engage arrestin-3, independent of their reward properties.

Here, we have demonstrated that MOR engagement with both G protein and arrestin-3 improves the therapeutic utility of opioids by reducing the development of analgesic tolerance without exacerbating respiratory effects. In combination with other data that suggests arrestin-3 recruitment can reduce dependence [14, 45, 57], abuse liability and relapse [15], these findings indicate that new opioid development efforts should focus on identifying compounds that signal with a profile mimicking that of endogenous ligands, which engage both G protein and arrestin-3.

## Supplementary information

Supplemental Material
