# Supplementary material for: Pharmacological and genetic manipulations at the µ-opioid receptor reveal arrestin-3 engagement limits analgesic tolerance and does not exacerbate respiratory depression in mice
Source: Neuropsychopharmacology. 2021 Jul 13;46(13):2241–9. doi: 10.1038/s41386-021-01054-x (PMC8581001; doi:10.1038/s41386-021-01054-x)
Supplement: Supplementary file 1 — Supplemental Material [file 41386_2021_1054_MOESM1_ESM.pdf]

Table S1. Morphine-induced analgesia and respiratory depression in WT, Arr-3 KO and RMOR mice

Mouse strains	AD_{50} Values of morphine (mg/kg) (95% confidence intervals)			Respiratory rate (bpm) AUC ± S.E.M.	
	Day 1	Day 8	Tolerance Index (Fold shift)	Saline	Morphine
WT	4.6 (3.1-6.1)	12.5 (10.7-15.2)	2.7	29525 ± 1440	20398 ± 577 ****
Arrestin-3 KO	3.9 (3.7-4.2)	10.2 (8.9-11.7)	2.6	27185 ± 943	14974 ± 511 ****
RMOR	1.0 (0.9-1.1)	1.6 (1.5-1.7)	1.6	28029 ± 1121	19520 ± 695 ****

1. The AD_{50} values and their 95% confidence intervals were calculated from Figure 1 using Prism.
2. The doses of morphine for respiratory measurements were the AD_{80} values: 8 mg/kg (WT), 6 mg/kg (Arr-3 KO), and 1.5 mg/kg (RMOR).

* significantly different from saline using 2-way ANOVA with Tukey's multiple comparisons test,
**** = P <0.0001

Table S2. Peak respiratory depression by genotype

Mouse strains	% of Baseline at Maximum Inhibition.		
	Saline	Morphine	Dose (mg/kg)
C57B/L6	59.2 ± 2.8	41.6 ± 2.3**	8
Arrestin-3 KO	72.4 ± 3.9	38.4 ± 1.7****	6
RMOR	62.1 ± 5.5	46.4 ± 3.8	1.5
129SvJ	62.9 ± 2.8	60.7 ± 3.7	8

* significantly different from saline using 2-way ANOVA with Tukey's multiple comparisons test, ** = P <0.01,
**** = P <0.0001

Table S3. Effect of opioid agonists on arrestin-3 recruitment *in vitro*

Agonist	Arrestin-3 recruitment	
	EC50 (uM)	Emax (%)
DAMGO	0.7 (0.4 - 1.3)	100.0 (89.6 - 111.5)
Morphine	0.7 (0.5 - 1.0)	33.8 (32.4 - 36.3)****
Methadone	0.8 (0.5 - 1.2)	86.0 (79.7 - 92.9)
Fentanyl	0.2 (0.1 - 0.3)	94.9 (87.9 - 102.2)
Buprenorphine	ND	ND
Oxycodone	17.4 (9.2 - 40)****	78.9 (60.1 - 121.3)*
TRV130	0.6 (0.1 - 2.6)	2.2 (1.7 - 2.9)****

"ND" = Not detectable
* significantly different from DAMGO using one-way ANOVA with Dunnett's multiple comparisons test,
* = P <0.05, **** = P <0.0001
*arrestin-3 recruitment was measured in CHO cells stably expressing the μ-opioid receptor

Table S4. The effects of opioid agonists on analgesia, respiratory depression and tolerance to these effects

Agonists	Analgesia AD_{50} (mg/kg) (95% confidence intervals)			% of baseline Respiratory Rate AUC± S.E.M. (Baseline @ 20% for 80 min)			
	Day 1	Day 8	Fold shift	Day 1	Day 8	Ratio (1) (Day 1: Vehicle)	Ratio (2) (Day 8: Day 1)
Morphine	4.63 (3.1--6.1)	12.5 (10.7-15.2)	2.7	3178 ± 183****	3155 ± 129	0.63	0.99
Methadone	4.56 (4.0-5.2)	5.3 (4.7-6.1)	1.2	2238 ± 186****	1976 ± 146	0.44	0.88
Fentanyl	0.052 (0.044-0.059)	0.077 (0.069-0.087)	1.5	3144 ± 306****	3148 ± 251	0.62	1.00
Buprenorphine	0.22 (0.19-0.25)	0.66 (0.61-0.74)	3.0	4570± 235	3864 ± 233	0.90	0.85
Oxycodone	2.57 (2.2-2.9)	5.79 (5.0-6.9)	2.3	3688 ± 99***	3527 ± 198	0.73	0.96
TRV130	1.5 (0.13-1.85)	5.7 (5.3-6.1)	3.8	3472 ± 208****	3144 ± 161	0.65	0.91

1. Vehicle is saline for all except TRV130. The AUC ± SEM for saline is 5072 ± 266
2. Vehicle is 1.5% DMSO in saline for TRV130. The AUC ± SEM for 1.5% DMSO is 5311 ± 220.

* significantly different from vehicle using one-way ANOVA with Dunnett's multiple comparisons test, ** = P <0.01, *** = P <0.001, **** = P <0.0001

Table S5. Maximum respiratory depression by opioid agonists

Agonists	% of Baseline at Maximum Inhibition	Ratio (Drug: Vehicle)
Morphine	41.6 ± 2.3	0.70
Methadone	35.1 ± 2.3	0.60
Fentanyl	45.7 ± 5.2	0.77
Buprenorphine	64.6 ± 4.2	1.09
Oxycodone	47.7 ± 2.1	0.81
TRV 130	45.4 ± 2.7	0.85

1. Maximums were compared to all other drugs with an ordinary one-way ANOVA and Tukey's multiple comparisons test (ns.)

1. Vehicle is saline for all except TRV130. The peak respiratory depression ± SEM for saline is 59.2 ± 2.8.
2. Vehicle is 1.5% DMSO in saline for TRV130. The peak respiratory depression ± SEM for 1.5% DMSO is 53.2 ± 4.9.

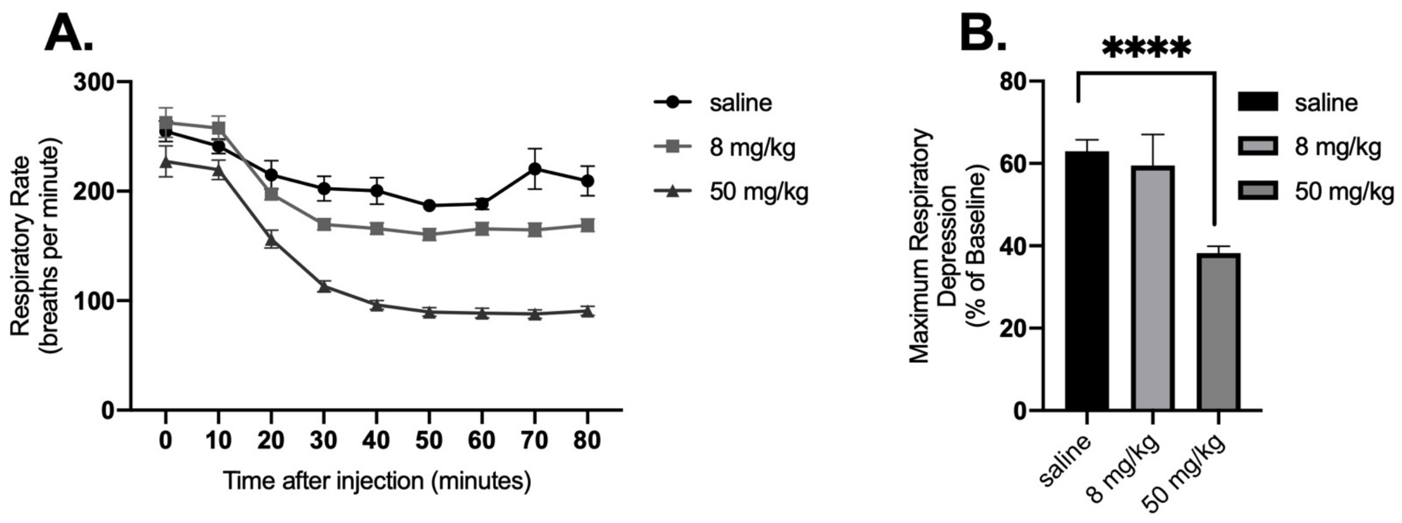

Figure S1. High morphine is required to produce respiratory depression in 129SvJ mice (A) Respiratory rates in 129SvJ mice with morphine versus saline. (B) Peak respiratory rates. (Ordinary one-way ANOVA, F = 20.79, P <0.0001).

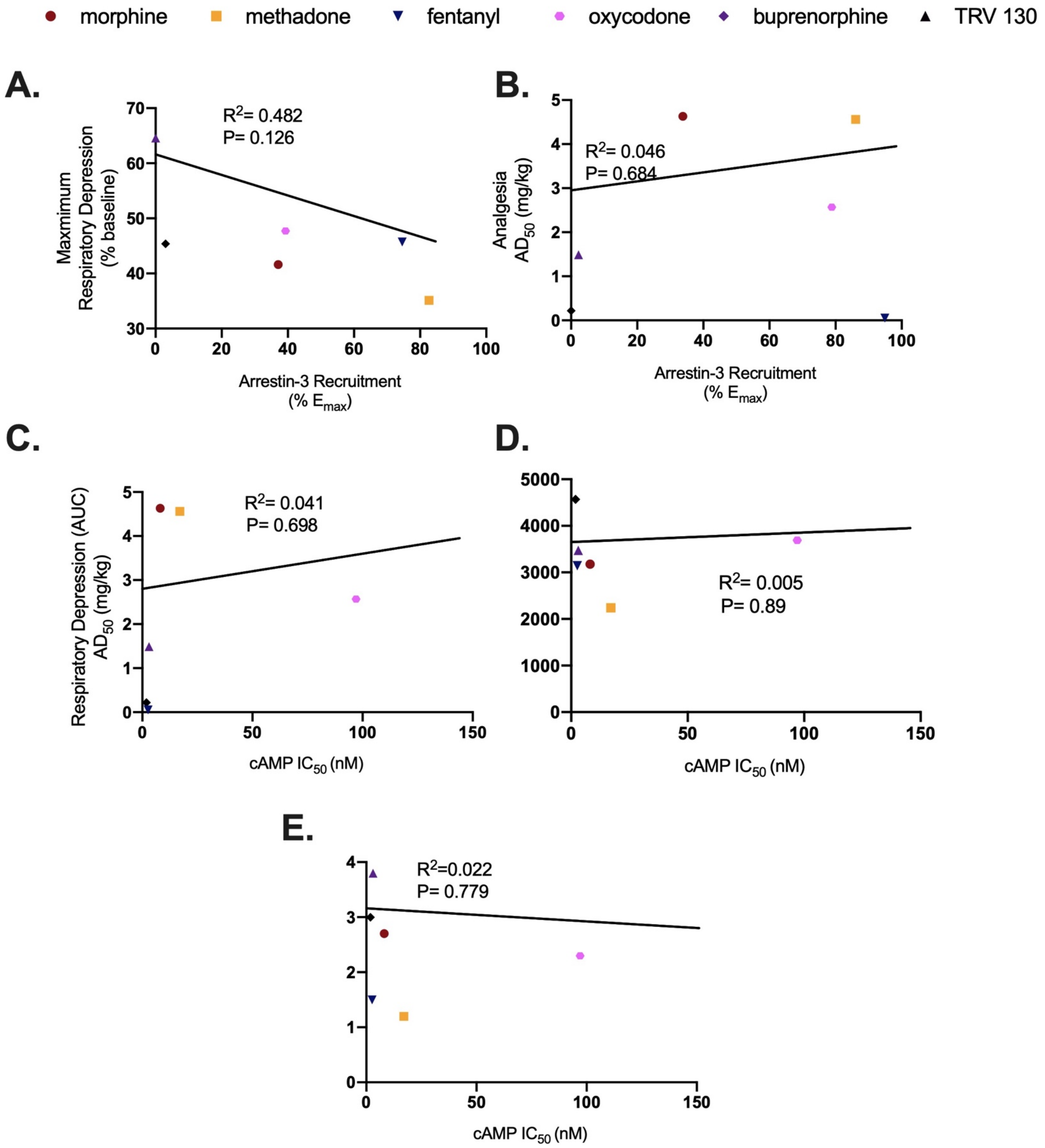

Figure S2. Correlation between the *in vitro* arrestin-3 recruitment or cAMP inhibition of the tested drugs and their respective *in vivo* respiratory depression and analgesic effects. Arr-3 recruitment plotted against peak respiratory inhibition (A) and analgesia (B). cAMP IC_{50} values plotted against AD_{50} values for (C) respiratory depression AUC and (D) analgesic tolerance AUC. (E) Analgesic tolerance index was the fold-shift of AD_{50} values calculated in Table 2. The respiratory depression AUC and peak inhibition were calculated from Figure 3D. Pearson correlation was used for the statistical analysis.

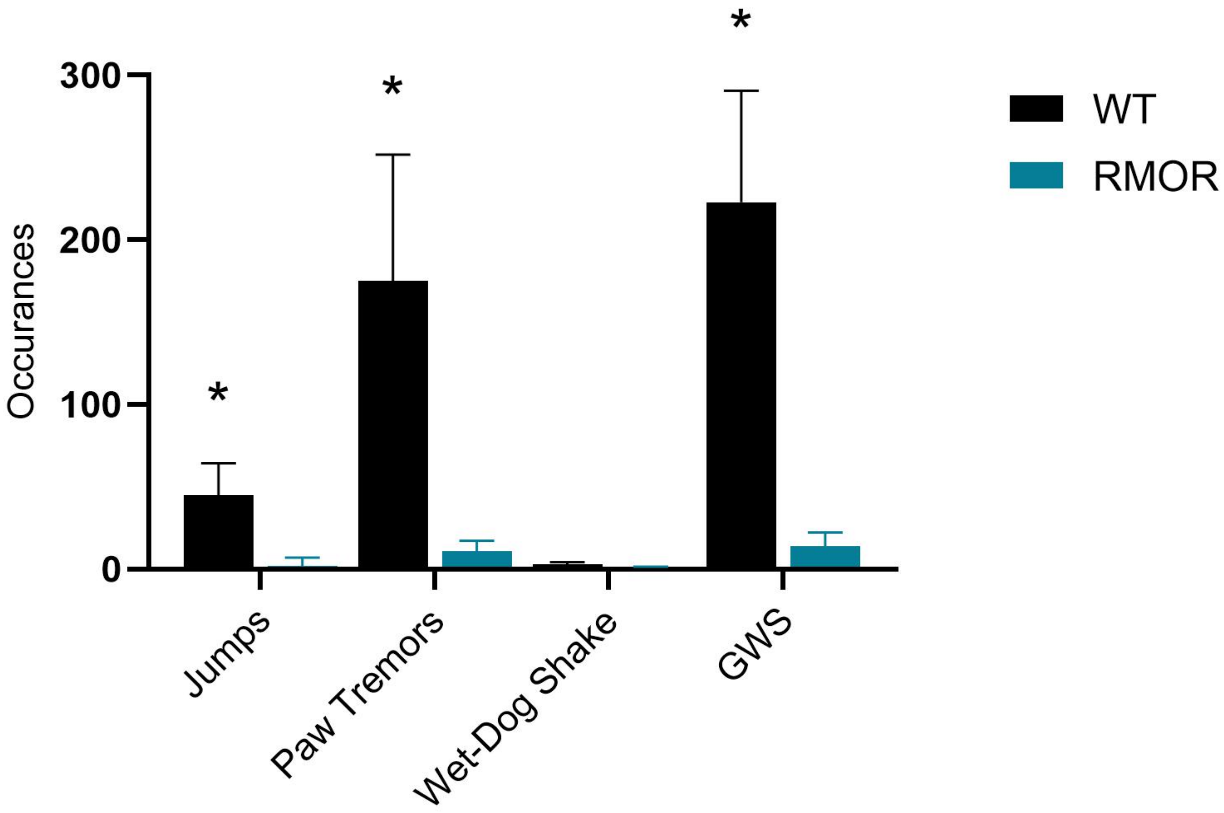

Figure S3. WT but not RMOR mice show dependence to morphine. Mice were treated with the AD_{80} dose of morphine (Figure 1) for 8 days. 30 minutes following the last dose of morphine, mice were administered naloxone (5 mg/kg) and withdrawal signs were scored by investigator blind to genotype. GWS, global withdrawal score is the sum of all withdrawal signs equally weighted. * = P <0.05 using two-tailed student's t-test.
